# Selective Blood Pressure Screening in the Young: Quantification of Population Wide Underestimation of Elevated Blood Pressure

**DOI:** 10.1155/2019/2314029

**Published:** 2019-05-02

**Authors:** Hannelore Neuhauser, Carolin Adler, Giselle Sarganas

**Affiliations:** ^1^Robert Koch Institute, Department of Epidemiology and Health Monitoring, Berlin, Germany; ^2^DZHK (German Centre for Cardiovascular Research), Partner Site, Berlin, Germany

## Abstract

Universal blood pressure (BP) screening in children and adolescents is questioned in prevention guidelines, while measuring blood pressure in the young in the context of overweight, obesity, or parental hypertension is promoted. This study quantifies with population data the underestimation of elevated blood pressure that would result from limiting BP screening only to those with overweight, obesity, or parental hypertension in the young. Selective screening was simulated with representative national health examination data from Germany (age 3-17, N=14,633, KiGGS0 study 2003-2006; age 18-39, N=1,884, DEGS1 2008-2011 study), with mean of two oscillometric measurements on one occasion; cutoffs for hypertensive BP in children were the 95th percentile using KiGGS percentiles, and for sensitivity analyses Fourth Report percentiles, in adults 140/90 mmHg; childhood overweight and obesity were classified according to the International Obesity Task Force and for adults as BMI ≥25 and ≥30 kg/m2. In 3-17-year-olds, different selective BP screening scenarios were simulated: screening only in those with obesity, overweight, parental hypertension, combination of overweight and parental hypertension, resulting in screening 5.6%, 20.0%, 28.5%, and 42.6% of the population and detecting 17.2%, 38.6%, 30.3%, and 58.2% of all hypertensive cases in the population. In conclusion our results show a large screening gap that would result from selective BP screening only in those with overweight, obesity, or parental hypertension.

## 1. Introduction

Blood pressure (BP) screening in children and adolescents is subject to an ongoing debate [1–8] and is recommended in hypertension guidelines [7–9] but questioned in prevention guidelines [2, 3]. BP screening in youth with obvious hypertension risk factors such as obesity has been suggested as a reasonable selective screening strategy [4] without reference to the implications of not screening everybody else. In young adults starting at age 18, universal BP screening is unequivocally recommended [10–12]. However, hypertension awareness rates in younger adults are rather low compared to older adults [13], suggesting that adherence to universal BP screening may be regarded as less important by the young adults themselves or by their healthcare providers.

Therefore, the aim of our study was to quantify the population wide underestimation of elevated blood pressure resulting from selective blood pressure screening in the young. Data from national health examination surveys in Germany across a wide age-range of 3-39-year-old participants were used to estimate how large the proportion of the population with undetected elevated BP would be if only those with overweight, obesity, or parental hypertension would get their BP measured.

## 2. Methods

### 2.1. Study Population

We used data of two recent health examination surveys in Germany: the German Health Interview and Examination Survey for Adults 2008-2011 (DEGS1) and the German Health Interview and Examination Survey for Children and Adolescents 2003-2006 (KiGGS). Both used a nationwide two-stage-clustered sample design. In the first step, communities were randomly selected on the basis of federal state and community type and size, and in the second step, a random selection of persons stratified for age and sex from the local population registers was performed [14, 15].

KiGGS included 14,835 children and adolescents aged 3 to 17 years from 167 communities, of whom 7,570 were boys and 7,265 were girls. DEGS1 comprised a total of 7,115 adults aged 18-79 years (men: 3,410; women: 3,705) from 180 communities, 1,912 of whom were aged 18-39 years (934 men, 978 women). The studies were approved by the ethical committee of Charite–University Medicine, Berlin, and by the Federal Commissioner for Data Protection and Freedom of Information. Informed written consent and assent were obtained from all adults and children aged 14 years or older as well as from parents of children younger than 14 years.

### 2.2. Measurements and Definitions

BP measurements followed a standardized protocol using an automated oscillometric Datascope Accutorr Plus device (Datascope Accutorr Plus, Mahwah, NJ, USA). Three readings of systolic (SBP) and diastolic BP (DBP), mean arterial pressure, and pulse rate were obtained in adults and the second and third measurements were averaged. In children, SBP and DBP from two readings were averaged. Participants sat upright on a height-adjustable chair with a backrest, the right forearm was resting on a table at the level of the heart, the elbow was slightly bent, the legs were not crossed, and the feet were placed firmly on the floor. For determination of the correct cuff size in adults, the circumference of the upper arm was measured half way between the acromion and the olecranon and one of three cuff sizes were chosen accordingly: cuff width 10.5 cm x cuff length 23.9 cm for arm circumferences of 21–27.9 cm, 13.5 × 30.7 cm for arm circumferences of 28–35.9 cm, and 17 × 38.6 cm for arm circumferences of 36–46 cm. In children, the cuff had to cover at least two-thirds of the upper arm length (from the axilla to the antecubital fossa) and 4 cuffs sizes were available: 6 × 12 cm, 9 × 18 cm, 12 × 23 cm, and 17 × 38.6 cm. The measurements were realized in 3-minute intervals (children: 2 minutes) after a 5-minute rest following a nonstrenuous part of the examination. A computer-assisted personal interview included questioning on current and past medical conditions and medication within the 7 days preceding the interview, and all medications were coded in accordance with the World Health Organization's (WHO's) Anatomic Therapeutic Chemical Classification System (ATC code).

Hypertensive BP in children and adolescents was defined as SBP or DBP ≥95th age- and height-specific percentiles derived from the KiGGS nonoverweight reference population according to German guidelines [16, 17]. Thereby, SBP ≥140 or DBP ≥90 mm Hg is classified as hypertensive irrespective of the BP percentile. For a sensitivity analysis, hypertensive BP in children was defined as SBP or DBP ≥95th age- and height-specific percentile as recommended by the ESH-2016 guideline [7] and corresponding to the Fourth Report percentiles [9]. Thereby also SBP ≥140 or DBP ≥90 mm Hg is classified as hypertensive irrespective of the BP percentile.

In adults, hypertensive BP wad defined as SBP ≥140 or DBP ≥90 mm Hg or on ATC-coded (C02, C03, C07, C08, C09) antihypertensive medication in case of known, physician-diagnosed hypertension [13].

Body height and weight measurements were performed by trained staff according to standardized protocols. Body height was measured to the nearest 0.1 cm using portable devices (Harpenden Stadiometer; Holtain Ltd, Crymych, United Kingdom). Body weight was recorded to the nearest 0.1 kg with the participants dressed only in underwear without shoes and using a calibrated scale (Seca, Birmingham, United Kingdom). Body Mass Index (BMI) was calculated as weight in kilogram (kg) divided by height in meter squared (m^2^). In adults, the WHO definition was used to categorize BMI groups as follows: normal-weight for BMI 18.5-24.9 kg/m^2^, overweight for BMI 25.0-29.9 kg/m^2^, and obesity for BMI ≥30.0 kg/m^2^. In children, overweight and obesity were defined according to the International Obesity Task Force (IOTF) [18] and for sensitivity analyses also according to the German references by Kromeyer-Hauschild [19].

History of parental hypertension was obtained as part of the personal medical interview in DEGS1 and by a telephone follow-up interview conducted from 2009 to 2012 in KiGGS with the question, “Has the mother or father of the study participant ever been diagnosed by a doctor as having hypertension or high BP?” Paternal hypertension status was unknown for 26.4% of participants aged 18-39 and 18.9% of those aged 3-17 years, and maternal hypertension status for 22.8% and 13.1%, respectively.

### 2.3. Analysis

1,884 adults aged 18-39 years had complete BP and BMI data (with 98.5% of DEGS participants aged 18-39 years). Complete data on BP and BMI and parental hypertension were available for 1,482 DEGS participants (with 77.5% of all participants aged 18-39 years). For children aged 3 to 17 years, there were 14,633 participants (with 98.6% of KiGGS participants aged 3 to 17 years) with complete data for BP and BMI. In each of the eight age and sex groups (3-6 years, 7-10 years, 11-13 years, and 14-17 years separately for boys and girls), there were 1,482-2,116 complete cases, corresponding to 95.9-99.6% of all KiGGS participants, respectively. 9,229 cases (with 62.2% of KiGGS participants being 3-17 years old) had complete data BP and BMI and parental hypertension.

We compared universal BP screening (i.e., measuring BP in the whole population) to selective screening scenarios, i.e., measuring BP only in those with overweight, obesity, or parental hypertension. We calculated common measures of diagnostic accuracy such as sensitivity, specificity, and positive and negative predictive value. Taking obesity as “test” and hypertensive blood pressure as gold standard, sensitivity was calculated as proportion of obese hypertensives out of all hypertensives and specificity as proportion of nonobese normotensives out of all normotensives. The positive predictive value was calculated as proportion of obese hypertensives out of all obese participants and the negative predictive value as proportion of nonobese normotensives out of all nonobese participants.

The KiGGS and DEGS1 data were weighted to the German reference population (KiGGS: 31 Dec 2004, DEGS1: 31 Dec 2010) with respect to age, sex, region, and nationality as well as type of municipality. All analyses were performed with the complex samples option in SPSS 20.0 to take into account the weighting and the correlation of the participants within a community.

## 3. Results

**Figure 1:**
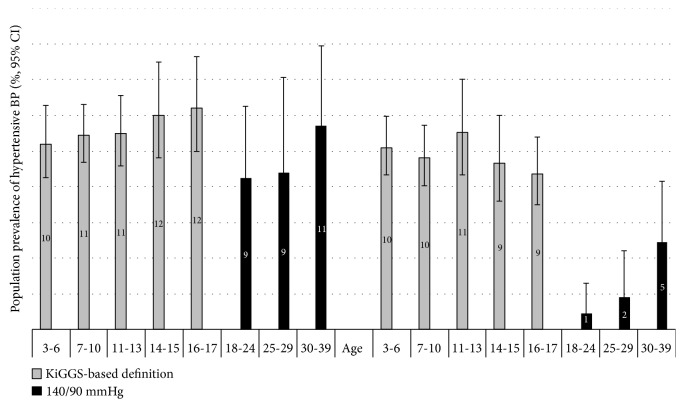
Prevalence of hypertensive BP by age groups.

The characteristics of the study population are shown in Table 1. According to KiGGS percentiles, which are based on a nonoverweight population from Germany, 10.5% of 3-17-year-old children had hypertensive BP (boys 11.2%, girls 9.9%) (Figure 1).

In adults aged 18 to 39 years the prevalence of hypertensive BP was 6.4% (9.9% in men and 2.9% in women).

In hypothetical selective screening scenarios that consist of measuring BP only in children and young adults with overweight, obesity, or parental hypertension, i.e., at increased risk of hypertension based on easily available clinical information, the proportion of the population that would be screened corresponds to the prevalence of the selection criterion for screening. The prevalence of overweight and obesity according to the German reference system, to IOTF, and to adult cutoffs as well as the prevalence of parental hypertension is shown in Table 1. For example, if the screening scenario would be to measure BP only in obese children (according to IOTF), then 5.6% would have their BP measured while 94.4% would not. When choosing overweight or parental history of hypertension as a criterion for measuring BP, then 42.6% of children would have their BP measured. In adults aged 18 to 39 years, obesity as a selection criterion for measuring BP would mean screening for high BP in less than one in six adults (13.8%), overweight one in four adults (26.7%), and parental history of hypertension almost half of adults (45.3%) (Table 1).

**Figure 2:**
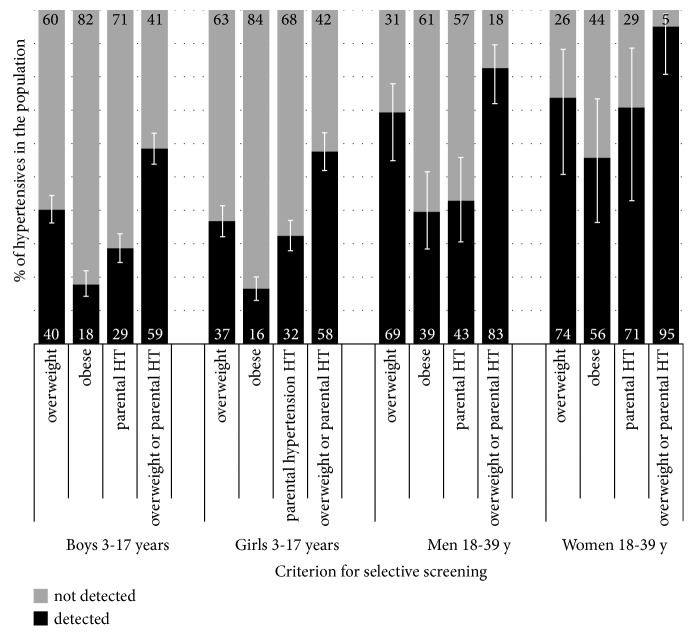
Sensitivity of overweight, obesity, and parental hypertension for detection of hypertensive blood pressure in children (KiGGS-based definition) and adults (140/90 mm Hg cutoff).

Although the presence of overweight, obesity, or parental hypertension generally increased the probability of having hypertensive BP, a large proportion of children with hypertensive BP did not belong to these subgroups. Therefore, choosing to measure BP only in children with overweight, or only those with obesity, or only those with parental hypertension would have low sensitivity (Table 2) for detecting hypertensive BP. Results are reported for overweight and obesity according to the IOTF references but are available upon request also for German references. For example, measuring BP only in obese children would detect 17.8% of all boys and 16.4% of all girls with hypertensive BP while well over 80% of children with hypertensive BP would go undetected since they are nonobese (Figure 2). Even when measuring BP in all children with overweight or parental hypertension, over 40% of cases with hypertensive BP would go undetected. In young adults, the sensitivity of selective screening in all those with overweight or parental hypertension was higher and reached 82.5% in men and 94.9% in women. For these results the German pediatric BP references were used but we report in the Supplementary Materials the analyses using the Fourth Report references.

In young adults aged 18-39 years, BP measurements restricted to overweight would identify 69.4% (95% CI 54.9-77.9) of men and 73.6% (95% CI 50.7-88.3) of women with actual hypertension (Figure 2). Screening restricted to obese adults would detect only 39.4% and 55.7% of hypertensive men and women, respectively.

Higher sensitivity of selective screening strategies was associated with lower positive predictive value (Table 2); e.g., the positive predictive value of overweight or parental hypertension in women aged 18-39 years for having hypertensive BP was only 4.2%, while the sensitivity was 94.9%.

Supplemental Table 1 shows the sensitivity of selective screening strategies stratified by sex and four age groups. Although there were differences between age groups, the general pattern of limited sensitivity already apparent for the overall age group of 3-17 years remained the same.

## 4. Discussion

Our analysis of population BP data quantifies the population wide underestimation of elevated blood pressure that would result from selective BP screening in the young, i.e., from routine BP measurements only in those with overweight, obesity, or parental hypertension. The importance of blood pressure in children has been frequently reported in the context of the obesity epidemic [2, 4, 20]. As a consequence, the frequency of BP measurement in ambulatory pediatric settings is higher in children with overweight and obesity [21]. Although it is common knowledge that hypertension does not occur only in the presence of overweight, obesity, or parental hypertension, there are only very scarce actual population data quantifying the relevance of the group that is missed if screening becomes selective [22].

Our analyses show that in children aged 3-17 years, selective screening of BP only in those with obesity would fail to detect 82% of cases with hypertensive BP in boys and 84% in girls aged 3-17 years. Even the most sensitive of the screening scenarios investigated, i.e., screening those with overweight or parental hypertension, would miss more than 40% of children with hypertensive BP. Of note, irrespective of the screening strategy, additional BP measurements on subsequent visits would have to follow before establishing the diagnosis of hypertension. In children, the prevalence of hypertensive BP measurements on one occasion is higher by approximately the factor 4 compared to the prevalence of hypertension based on hypertensive BP values on three occasions [23]. While we screened for hypertensive BP on one occasion, a Swiss study with 6th grade students investigated screening for sustained hypertensive BP over three separate visits and found only slightly higher sensitivities of targeted screening based on overweight, obesity, and parental hypertension (including combinations). For example, if only obese children would participate in the screening, hypertensive BP on one occasion would be detected only in 20.6% of 11-13-year-old children according to our study, and hypertension defined as sustained raised BP on three occasions would be detected in 24.8% of 10-14-year-old children according to the Swiss study [22]. The highest sensitivity was achieved when screening all children with overweight or parental hypertension (51.7% in our study and 64.6% in the Swiss study); however, a high proportion of children with raised BP or, respectively, with hypertension remain undetected. In accordance with these findings, sustained hypertensive BP was examined in a 6-year tracking BP study, where the positive predictive value of obesity at baseline for a hypertensive BP measurement six years later was 36% in 3- to 10-year-olds and 50% in 11- to 17-year-olds and for children with parental hypertension was 18% and 21%, respectively [24].

Strengths of our analysis include the large and population-based sample with BP data based on highly standardized measurements with one of the few oscillometric devices validated in children [25, 26]. In the validation study with children aged 5-15 years the oscillometric Datascope SBP was 0.9±4.33 mmHg lower compared to mercury sphygmomanometer measurements, DBP 1.2±6.48 lower, and passed the International Protocol of the European Society of Hypertension adapted for validation in children [25, 27]. It is a strength that our BP data are means of two measurements taken on one occasion, but it is a limitation that we could not measure BP on several occasions. Moreover, even with validated oscillometric devices, guidelines recommend confirmation by auscultatory BP measurement [7] which was not possible in our study and represents a limitation. An additional limitation is the reduced sample size for the analyses concerning parental hypertension. However, the bias resulting from this incomplete information is unlikely to change the conclusion that parental hypertension is not a sufficient criterion for deciding who should or should not get BP measurements.

A quantitative method to determine the optimal cutoff value for a screening test is the Receiver Operating Characteristic Curve Analysis. Applied to our study, this would mean to determine the optimal BMI cutoff for identifying children and young adults with hypertensive BP. However, it already becomes evident from the two BMI cutoffs used in the analysis (defining overweight and defining obesity) that although the sensitivity increases when the BMI cutoff is lowered, even when using the overweight cutoff instead of the obesity cutoff, sensitivity in absolute terms remains moderate and is insufficient to serve as a screening test.

In summary, our analysis shows with population data that selective BP screening only in children with obvious hypertension risk factors such as overweight or parental hypertension would leave large proportions of children with elevated BP undetected. This conclusion holds both for children and young adults and for elevated BP defined by KiGGS percentiles as well as by Fourth Report percentiles.

## Figures and Tables

**Table 1:** Hypertensive blood pressure, overweight, obesity, and parental history of hypertension in 3-39-year-olds in Germany.

	Total		Male		Female	
*Children and adolescents 3-17 years*	*n*		*n *		*n*	

KiGGS 2003-2006 participants	14835		7570		7265	
Participants with complete data on BP and BMI	14633		7488		7145	
Participants with complete data on BP, BMI and parental hypertension	9229		4733		4496	

	* (%)*	* 95 %-CI *		*95 %-CI *		*95 %-CI*

hypertensive BP (KiGGS percentiles)	10.5	(9.7-11.5)	11.2	(10.2-12.3)	9.9	(8.9-10.9)
hypertensive BP (Fourth Report)	6.7	(6.1-7.4)	7.3	(6.5-8.2)	6.1	(5.4-7.0)
overweight: German reference	16.0	(15.1-16.8)	15.8	(14.7-17.1)	16.1	(15.1-17.2)
overweight: IOTF	20.0	(19.1-20.9)	19.8	(18.5-21.2)	20.2	(19.0-21.4)
obese: German reference	7.1	(6.5-7.7)	6.7	(6.0-7.6)	7.4	(6.6-8.2)
obese: IOTF	5.6	(5.1-6.2)	5.6	(4.9-6.4)	5.7	(5.0-6.4)
parental hypertension	28.5	(27.3-29.8)	28.9	(27.4-30.5)	28.1	(26.7-29.6)
overweight (IOTF) or parental hypertension	42.6	(41.3-43.8)	43.0	(41.3-44.7)	42.1	(40.6-43.7)
obese (IOTF) or parental hypertension	32.4	(31.1-33.7)	32.8	(31.1-34.5)	32.1	(30.5-33.7)

*Adults 18-39 years*	*n*		* n*		*n*	

DEGS1 2008-2011 participants	1912		934		978	
Participants with complete data on BP and BMI	1884		923		961	
Participants with complete data on BP, BMI and parental hypertension	1482		688		794	

	* (%)*	* 95 %-CI *		*95 %-CI *		*95 %-CI*

hypertensive BP	6.4	(5.2-7.9)	9.9	(7.7-12.6)	2.9	(1.9-4.5)
overweight	26.7	(24.5-29.0)	32.8	(29.4-36.4)	20.4	(17.6-23.4)
obese	13.8	(12.1-15.7)	14.6	(12.1-17.4)	13.0	(10.8-15.5)
parental hypertension	45.3	(42.5-48.1)	42.4	(38.0-46.9)	48.0	(44.1-52.0)
overweight (IOTF) or parental hypertension	65.1	(62.1 67.9)	66.9	(62.4 71.0)	63.4	(59.4 67.1)
obese (IOTF) or parental hypertension	52.0	(49.0 55.0)	50.4	(45.9 54.9)	53.6	(49.5 57.7)

KiGGS: German Health Interview and Examination Survey in Children and Adolescents; DEGS1: German Health Interview and Examination Survey for Adults.

BP: blood pressure; BMI: Body Mass Index; IOTF: International Obesity Task Force (cut points correspond to an adult BMI of 25 (overweight) or 30 (obesity)).

Hypertensive BP: SBP or DBP ≥95th percentile or ≥140/90 mm Hg.

**Table 2:** Sensitivity, specificity, and positive and negative predictive values of selective screening scenarios for detecting hypertensive blood pressure.

Selective screening based on	Sensitivity (95% CI)		Specificity (95% CI)		Positive predictive value (95% CI)		Negative predictive value (95% CI)	
*Boys 3-17 years*								
overweight	40.2	(36.2-44.4)	82.8	(81.5-84.0)	22.8	(20.2-25.6)	91.7	(90.7-92.5)
obesity	17.8	(14.4-21.9)	95.9	(95.3-96.5)	35.6	(29.8-41.9)	90.3	(89.2-91.2)
parental hypertension	28.6	(24.4-33.1)	71.0	(69.3-72.7)	11.2	(9.4-13.2)	88.6	(87.1-90.0)
overweight or parental hypertension	58.6	(53.9-63.1)	59.0	(57.2-60.7)	15.4	(13.6-17.4)	91.8	(90.5-92.9)
obesity or parental hypertension	42.0	(37.1-47.1)	68.4	(66.6-70.2)	14.5	(12.5-16.8)	90.3	(88.8-91.5)

*Girls 3-17 years*								
overweight	36.7	(32.2-41.4)	81.6	(80.4-82.8)	17.9	(15.5-20.6)	92.2	(91.1-93.1)
obesity or parental hypertension	16.4	(13.2-20.2)	95.5	(94.9-96.1)	28.6	(23.4-34.5)	91.3	(90.2-92.2)
parental hypertension	32.3	(27.9-37.1)	72.4	(70.8-73.9)	11.5	(9.5-13.7)	90.6	(89.1-91.9)
overweight or parental hypertension	57.6	(51.9-63.1)	59.6	(57.9-61.2)	13.6	(11.7-15.7)	92.7	(91.2-94.0)
obesity or parental hypertension	43.3	(37.8-49.0)	69.2	(67.5-70.8)	13.5	(11.3-16.0)	91.7	(90.2-92.9)

*Men 18-39 years*								
overweight	69.4	(59.4-77.9)	55.0	(51.0-59.0)	14.4	(11.0-18.7)	94.3	(91.5-96.2)
obesity	39.4	(28.4-51.6)	88.1	(85.0-90.6)	26.6	(18.0-37.5)	93.0	(90.5-94.9)
parental hypertension	42.8	(30.7-55.9)	57.7	(52.8-62.4)	11.2	(7.8-15.8)	89.0	(84.1-92.5)
overweight or parental hypertension	82.5	(72.0-89.7)	35.1	(30.5-40.0)	13.7	(10.4-17.9)	94.2	(89.5-96.8)
obesity or parental hypertension	65.4	(50.7-77.6)	51.5	(46.7-56.3)	14.4	(10.6-19.3)	92.3	(87.3-95.4)

*Women 18-39 years*								
overweight	73.6	(50.7-88.3)	67.9	(64.5-71.1)	6.4	(3.9-10.3)	98.9	(97.3-99.5)
obesity	55.7	(36.4-73.4)	88.3	(85.7-90.5)	12.4	(7.0-20.9)	98.5	(97.3-99.2)
parental hypertension	70.7	(42.8-88.6)	52.6	(48.6-56.6)	4.1	(2.3-7.1)	98.4	(95.8-99.4)
overweight or parental hypertension	94.9	(80.6-98.8)	37.5	(33.7-41.6)	4.2	(2.5-6.9)	99.6	(98.5-99.9)
obesity or parental hypertension	94.9	(80.6-98.8)	47.6	(43.4-51.8)	4.9	(2.9-8.1)	99.7	(98.8-99.9)

Overweight and obesity according to International Obesity Task Force cut points corresponding to an adult BMI of 25 (overweight) or 30 (obesity).

Hypertensive blood pressure: age 3-17 years: SBP or DBP ≥95th KiGGS percentile or ≥140/90 mm Hg; age 18-39 years ≥140/90 mm Hg.

## Data Availability

The authors confirm that some access restrictions apply to the data underlying the findings. The data set cannot be made publicly available because informed consent from study participants did not cover public deposition of data. However, the minimal data set underlying the findings is archived in the Data Centre at the Robert Koch Institute (RKI) and can be accessed by all interested researchers. On-site access to the data set is possible at the Secure Data Centre of the RKI. Requests should be submitted to the Robert Koch Institute, Berlin, Germany (e-mail: datennutzung@rki.de).

## Supplementary Materials

Supplementary Materials Supplemental Table 1: sensitivity of selective screening scenarios for detecting hypertensive blood pressure (KiGGS-based definition). Supplemental Table 2: sensitivity, specificity, and positive and negative predictive values of selective screening scenarios for detecting hypertensive blood pressure (Fourth Report-based definition).
